# Association Between Maternal HIV Stigma Among South Indian Mothers Living with HIV and the CD4 Count of Children Living with HIV

**DOI:** 10.1007/s10461-021-03537-w

**Published:** 2021-12-11

**Authors:** Valerie PhamDo, Adeline M. Nyamathi, Maria L. Ekstrand, Sanjeev Sinha, Kartik Yadav, Sanghyuk S. Shin

**Affiliations:** 1 Sue & Bill Gross School of Nursing, University of California, Irvine, CA USA; 2 Center for AIDS Prevention Studies, Department of Medicine, University of California, San Francisco, CA USA; 3 Division of Medical Informatics, St. John’s Research Institute, Bangalore, India; 4 Department of Medicine, All India Institute of Medical Sciences, New Delhi, India

**Keywords:** Maternal HIV stigma, Discrimination, Seroconcordant, Child CD4

## Abstract

HIV stigma takes a multidimensional toll on a mother’s ability to care for herself and subsequently may impact her ability to care for her child, particularly when mother and child are seroconcordant. A cross-sectional analysis was conducted to examine the association between maternal HIV stigma and child CD4 count in rural India. We assessed 108 mother–child dyads and found that a one-unit increase in community stigma fear decreased child CD4 count by 352 cells (95% CI = − 603, − 102), highlighting the need to develop a better understanding of the consequences of HIV-related stigma on the compounded burden of care in households where mother and child both live with HIV.

## Introduction

### HIV Epidemic Among Children and Women

Globally, an estimated 3 million children (≤ 19 years) are living with HIV [1]; In India, this figure is approximately 120,000, the highest number reported for South Asia [1]. The primary mode of pediatric HIV transmission in resource-limited settings is perinatal transmission, from a mother living with HIV to her child during pregnancy, delivery, or breastfeeding [2, 3]. Hence, the extent of pediatric HIV infection is directly linked to the epidemiology of infection among women of reproductive age [4].

Biological vulnerabilities [5, 6], social norms, gender inequality [7], and cultural taboos about sexuality intersect to create structured disadvantages that lead to serious health implications for women, obstructing the ability of female children and women to safeguard their reproductive health [8, 9]. Structural inequalities [7], complicated by power structure and social hierarchy, lead to barriers that limit access to healthcare and make India’s children and women disproportionately at risk for HIV infection [10, 11]. Studies have shown that HIV stigma and discrimination adversely impact the physical, mental, and social well-being of women living with HIV, leading to an internalization of that stigma (i.e., acceptance of damaging self-image and fear of discrimination from HIV serostatus disclosure) [12]. Despite much progress in HIV research and treatment that make viral suppression possible for people living with HIV (PLWH), HIV stigma remains a major cause of physical, psychological, and social damage for PLWH, particularly for women living with HIV (WLWH) [13].

### HIV and Stigma

HIV stigma has “social and moralistic connotations” [14] that involve negative attitudes, preconceived ideas, and undesirable beliefs about PLWH, often due to the tendency to blame PLWH for their infections; it is the prejudice that comes with categorizing an individual as part of a group that is deemed to be socially unwanted [15]. Current research [16] shows the main drivers of HIV stigma are pre-existing prejudices towards key populations at higher risk of HIV infection, such as people who inject drugs, transactional sex workers, inmates, transgender individuals, and men who have sex with men. Additional factors [16, 17] include misconceptions about casual transmissions, lack of personal experiences with PLWH, irrational fear of infection, and blame. While HIV continues to be a highly scorned illness [18] and the stigma associated with HIV continues to be a public health challenge globally, the impact of HIV-related stigma is worse in settings where HIV-related discrimination is gendered in nature. In their 2001 landmark study of HIV and AIDS in India, Bharat et al. [17] reported the neglect and maltreatment of WLWH by their spouses and in-laws. Specifically, Bharat and colleagues reported that WLWH were less likely than men to seek testing, received poorer quality care than their husbands, more likely to take care of their seropositive husbands, and often blamed for their husbands’ infection by not controlling their husbands’ extramarital desires.

HIV stigma has multiple behavioral consequences for PLWH [19]. Fear of stigma leads to care delays, negatively impacting disease management, and adversely affecting health outcomes [20–24]. Specifically, in India, HIV-related stigma is found to be associated with care seeking delays, both directly (i.e., when an individual chooses to not seek care for fear of mistreatment by healthcare staff) and indirectly (e.g., when an individual chooses to not disclose HIV status to family and consequently is unable to seek financial help for medical expenses) [22]. In India, the association between internalized stigma and low treatment adherence, and the association between internalized stigma and low quality of life were mediated by lack of disclosure and social support [25, 26]. In terms of psychological challenges, it is well documented that stigma affects the emotional wellbeing and mental health of PLWH [27–29]. A systematic review and meta-analyses of the global literature published between 1996 and 2013 confirmed significant associations between HIV-related stigma and higher rates of depression, lower social support, lower usage of health and social services, and lower levels of adherence to antiretroviral treatment; these associations held up irrespective of cultural identity, sexual orientation, and sexual risk behaviors [30].

Stigma takes a multifaceted toll on the physical and psychological health of the maternal figure that, in turn, may lead to negative ramifications for the child [31–35]. Therefore, understanding the relationship between mothers’ perceived HIV-related stigma and the health among their children is crucial [36], especially when both mother and child live with HIV, which may compound consequences of HIV-related stigma. In India, maternal-child HIV research is focused primarily on prevention of mother-to-child transmission [37], with few studies exploring mother and/or caregiver-child dyads outside the context of transmission prevention [38], and even fewer studies investigating mother–child HIV seroconcordant dyads [39]. Borrowed from epidemiological studies regarding HIV transmission risk in intimate relationships [40] where seroconcordance describes relationships where couples are both HIV positive, we extend the concept of seroconcordance beyond intimate couples to include HIV-positive mother–child dyads. While there is a growing body of evidence regarding the role of HIV stigma on the health of WLWH, not much is known whether the same barriers that prevent a mother from accessing healthcare also impact her HIV infected child.

To our knowledge, no other published study has explored how maternal HIV-related stigma is associated with the CD4 count of children living with perinatally-acquired HIV (CLWPH) in low- and middle-income countries. While the multidimensional adverse consequences of HIV-related stigma on mothers are well-documented, the present paper explores outcomes among their children. In this present study, we sought to explore the seroconcordant dyad, in the context of whether different types of maternal HIV-related stigmas correlate with the child’s immune function, expressed as CD4 count.

## Methods

### Design and Sample

This cross-sectional secondary analysis used data from a quasi-experimental trial funded by the National Institute of Mental Health (R01MH098728) that investigated the impact of a nurse-led ASHA (Accredited Social Health Activist)-supported behavioral and nutritional intervention among WLWH in rural Nellore and Prakasam, India between April 2014 and November 2016 [41, 42]. Flyer-based recruitment was utilized to engage participants from 16 community health centers and primary health clinics.

The parent study enrolled 600 WLWH between 18 to 50 years of age, diagnosed with HIV, receiving treatment for at least three months prior, have CD4 T-cell counts > 100 cells/mm^3^, and have a child between 3 to 8 living with them. If there were more than one child, only the eldest child was included in the parent study. Women who previously participated in the ASHA-Life pilot study were excluded. Data were collected by trained ASHAs during face-to-face interviews using a baseline questionnaire. Blood samples were collected for CD4 count determination. Full study details, including design, sample, setting, and procedures were previously described elsewhere [41, 42]. For this present study, the sample included only seroconcordant dyads, where mother and child must both be diagnosed with HIV, reducing the parent study of 600 to a subsample of 109 mother–child dyads.

### Measures

A baseline questionnaire collected maternal sociodemographic information, CD4 cell counts of mother and child, and mother’s HIV-related stigma variables. Sociodemographic factors collected from mothers included age, residence (Nellore or Prakasam), marital status (married, separated/ divorced, or widowed), number of children, gender of children, age of children, monthly household income (500–1200 rupees, 1500–2300 rupees, or 2400–3200 rupees), religion (Hindu, Muslim, or Christian), and educational attainment (none, 1–9 years, or ≥ 10 years). CD4 cell counts from mother and child were collected using flow cytometry. Blood samples were sent to the Nellore District Hospital diagnostic laboratory and the Act Diff Coulter Analyzer was used for CD4 determination [41, 42]. The absolute numbers of CD4 cells were obtained by multiplying percent CD4 by total white blood cell count [41, 42]. The outcome of interest for this secondary analysis was the CD4 count of CLWPH, predicted by maternal HIV stigma variables, discussed below.

#### HIV-Related Stigma Variables

Stigma variables measured included mother’s stigma fears (fear of stigmatization upon HIV status disclosure to different social groups), stigma-avoidant coping, enacted stigma, internalized stigma, and vicarious [43] stigma. The scales used in the questionnaire were adapted to fit the current sample. For each stigma measure, a mean scale score was calculated for each category, except for enacted stigma, in which the total index score was constructed by calculating the sum of responses. Cronbach’s alpha for each scale was calculated based on 109 dyads in this analysis.

#### Stigma Fears

Developed and previously used in India [26], this instrument used five subscales (family, friends, healthcare workers, coworkers, and community) to assess the degree (very worried, somewhat worried, a little worried, or not at all worried) of fear of discrimination/stigmatization upon HIV serostatus disclosure to social groups. For this study, we omitted the work subscale from further analysis, as 100% of our sample indicated “not applicable” to the last half of the scale items (withholding of promotions, forced relocation, mental harassment, and forced voluntary retirement).

##### Family

This 6-item subscale measured the degree of worry of consequences driven by stigmatization upon HIV/AIDS status disclosure to family, such as physical abuse, verbal abuse/humiliation, family members keeping their distance, rejection by family, forced to leave the family home, and denial of property rights/inheritance of property. Previously a 7-item scale, this instrument was reduced to 6 items by omitting fear of divorce, as 56% of our sample was not married. Cronbach’s alpha was 0.59 for the full 7-item scale but improved to 0.80 after removing the irrelevant item.

##### Friends

This 6-item subscale asked mothers to rate their degree of worry of the following stigma consequences upon status disclosure to friends, such as rejection, labeled an “HIV/AIDS patient,” indirect taunts/humiliation in front of others, worried about friends divulging mother’s HIV status to others, verbal abuse/humiliation, and accused of immoral behavior. Reliability of this scale was 0.86.

##### Healthcare Workers

This 5-item subscale asked mothers to rate their degree of worry of the following discriminatory consequences upon HIV/AIDS status disclosure to health care workers, such as health care workers treating PLWH worse than other patients, labelled an “AIDS patient,” verbal abuse/humiliation, negligence in treatment, and health care workers taking unnecessary precautions (e.g., wearing a mask, double gloves) when caring for PLWH. Reliability of this scale was 0.80.

##### Community

This 12-item subscale, henceforth referred as “community stigma fear,” measured mother’s degree of worry of stigmatizing consequences upon HIV status disclosure to community members, such as expulsion from home by community members/leader, avoidance at social functions, avoidance at religious functions, labelled an “AIDS patient,” physical abuse, verbal abuse/humiliation, forceful eviction from the main village, forced funeral services on the outskirts of the village or community, denial of proper funeral rites, denial of school for her children, and siblings or children not able to find an alliance for marriage. Internal consistency for this scale was 0.90.

#### Stigma-Avoidant Coping

Stigma-avoidant coping was measured using a 9-item “disclosure avoidance” [43] questionnaire to assess the frequency (never, rarely, sometimes, or often) of coping behaviors PLWH use to conceal their HIV/AIDS diagnosis, such as describing their HIV/AIDS diagnosis as another disease, making up reasons for medical visits, using medical/pharmacy services outside the local village/community, concealing HIV/AIDS serostatus from health care workers, not taking or hiding ART medications in the presence of others, and avoiding status disclosure to family and friends to prevent them from telling others. Internal consistency for this scale was 0.87.

#### Enacted Stigma

Developed by Ekstrand et al. [21] for an Indian PLWH cohort and modified to fit our population [18, 22, 23], this variable measured overt acts of discrimination using a 10-item scale designed to capture specific discriminatory acts mothers experienced due to their HIV infection [44]. Mothers responded in a yes/no format to whether they experienced overt acts of discrimination, such as forced to move out of their home, asked to not touch or care for children, looked at differently, threatened physically, told not to share food/utensils with family, and denied medical care/hospital services. Internal consistency for this scale was 0.73.

#### Internalized Stigma

Internalized stigma describes when a person living with HIV absorbs negative cultural beliefs, feelings, or thoughts about PLWH, leading to feelings of shame, fear of disclosure, isolation, and despair [45]. Specific to our population [21, 29], this subscale gauged the degree to which mothers believed they deserved to be shunned or shamed based on their HIV/AIDS diagnosis [43]. Modified from a 10-item scale for PLWH in India [46], our 8-item instrument asked mothers to rate the extent (not at all, a little, a fair amount, or a great deal) they feel they should avoid the following scenarios as a person with HIV/AIDS: holding an infant, feeding children, sharing dinnerware, cooking, and visiting others. The next three items pertain to the extent they agree with the following scenarios: brought shame to the family, paying for karma or sin, and feeling disgusting. Two items (diagnosed with HIV/AIDS because of wrong behaviors and feeling guilty about having HIV/AIDS) were eliminated for low correlation. Cronbach’s alpha for our modified 8-item scale was 0.81.

#### Vicarious Stigma

Based on the observational learning literature, vicarious stigma [43] describes a change in behavior after witnessing or hearing  of consequences happening to others, even without personal experience [47]. This 10-item scale asked mothers how often (never, rarely, sometimes, or frequently) they heard stories about the mistreatment of PLWH, such as PLWH forced by family members to leave their home, ostracization by the village/community, family avoiding or refusing care of PLWH, denial of hospital services, mistreatment by hospital workers due to positive HIV serostatus, and hospital staff making PLWH’s infection publicly known by marking HIV/AIDS on their medical records. Reliability for this scale was 0.91.

### Statistical Analysis

The overall purpose of this analysis was to determine the association between maternal HIV stigma and the CD4 count of children living with perinatally-acquired HIV. We used linear regression to investigate whether mother’s fear of being stigmatized upon status disclosure to different social groups, stigma-avoidant coping, enacted stigma, internalized stigma, and vicarious stigma were correlated with her child’s CD4 count. Characteristics of mother participants considered for possible confounding were age, marital status, number of children, income, CD4 count, religion, and educational attainment. Given that our sample size (n = 109) did not allow for control of all demographic variables, only variables associated with the outcome in bivariate models with p-value < 0.20 were included in the final model. Maternal CD4 count and child BMI were not included in the final model to prevent over-adjustment bias [48]. We omitted maternal CD4 count and child BMI, as they could be mediators of the association between maternal HIV stigma and child CD4 count. Given the cross-sectional study design, we could not determine the temporality between these variables.

Using univariate frequency distribution methods, descriptive analysis was conducted for all relevant categorical demographic variables and possible correlates. A correlation matrix was created for pair-wise comparison between each variable. To analyze the relationship between child CD4 count and maternal HIV stigma correlates, linear regression analysis was conducted. Initial base model building included all HIV-related stigma variables, regardless of p-value. Starting with the set of stigma fears, each stigma fear subscale was added into the model one at a time. All stigma variables were analyzed as continuous variables. To control for confounding, additional variables (religion and educational attainment) were added to the full model because their *p*-values (< 0.2) were significant in the univariate analysis. Dummy coded variables were religion and educational attainment. The final model was verified for normality of residuals, multicollinearity, and heteroskedasticity. To estimate the internal consistency reliability of the stigma measures, Cronbach’s alpha was utilized. All analyses were conducted in STATA 15.0. The standard used for statistical significance was a 2-tailed *p* < 0.05.

## Results

Tables 1 and 2 contain descriptive statistics for 109 dyads, after dropping dyads without seroconcordant diagnoses from the parent sample. Mothers were between 21 and 48 years of age, but the majority (56.9%) were between 31 and 40 (M = 33, SD = 6.02). They had an average CD4 count of 459 cells/mL. The majority (88%) resided in Nellore with the following civil statuses: 49.5% widowed, 43.1% married, and 7.3% divorced/separated. In terms of income, the average monthly household income ranged from 500 to 3200 Indian rupees (i.e., currency conversion of ~ $6.73 to ~ $43.06 US dollars) (M = 1998 rupees, SD = 695.62), and half (50.5%) reported a monthly household income between 1500 and 2300 rupees. In terms of religious affiliation, most mothers identified as belonging to Hindu faith (67.9%). As for educational attainment, 45.9% of mothers reported having no formal education, 43.1% reported having 1–9 years of education, while 11% had ≥ 10 years of educational attainment.

**Table 1:** Characteristics of mothers and children living with HIV/AIDS (n = 109)

Mother’s characteristic	Mean (SD) or n (%)	Child’s CD4 count Mean (Std. Dev.)	Pearson’s coef r	Test statistic	p-value
*Residence*				*t*-value = 0.23	0.816
Nellore	96 (88.1%)	767 (289.4)			
Prakasam	13 (11.9%)	748 (115.8)			
*Age*				F-value = 1.31	0.274
21–30	38 (34.9%)	817 (321.4)			
31–40	62 (56.9%)	728 (253.4)			
41–50	9 (8.26%)	794 (152.5)			
*Marital status*				F-value = 0.03	0.971
Married	47 (43.1%)	770 (273.9)			
Divorced/separated	8 (7.3)%	773 (414.8)			
Widowed	54 (49.5%)	758 (255.5)			
*Number of children*				F-value = 0.58	0.559
1	31 (28.4%)	782 (328.5)			
2	60 (55.1%)	774 (238.5)			
3	18 (16.5%)	701 (290.8)			
*Monthly household income (rupees)*				F-value = 1.06	0.349
500–1200	17 (15.6%)	679 (310.1)			
1500–2300	55 (50.5%)	771 (318.1)			
2400–3200	37 (33.9%)	794 (163.2)			
*Religion*				F-value = 3.47	0.0348
Hindu	74 (67.9%)	724 (257.7)			
Muslim	9 (8.3%)	751 (212.1)			
Christian	26 (23.9%)	885 (310.8)			
*Educational attainment*				F-value = 1.77	0.176
None	50 (45.9%)	729 (298.5)			
1–9 years	47 (43.1%)	767 (245.2)			
≥ 10 years	12 (11.0%)	894 (256.8)			
*CD4 count (cells/mL)*	459 (286.5)		0.2135	*t*-value = 2.26	0.026

Child’s characteristic	Mean (SD) or n (%)	Child’s CD4 count Mean (Std. Dev.)	Pearson's coef r	Test statistic	p-value
*Gender*				t-value = − 0.85	0.4
Male	55 (50.5%)	742 (262)			
Female	54 (49.5%)	787 (286.8)			
*Age*				t-value = 0.84	0.40
3–5	15 (13.8%)	820 (328.1)			
6–8	94 (86.2%)	756 (265.6)			
*BMI*	15 (2.25)		0.2016	t-value = 2.13	0.036

**Table 2:** Stigma variables of mothers living with HIV (n = 109)

Stigma scale	Mean	Coef r	*t*-value	*p-*value
Stigma fear-family*	2.49	0.0458	0.47	0.638
Stigma fear-friends	2.89	− 0.1551	− 1.62	0.107
Stigma fear-healthcare	2.8	− 0.0796	− 0.83	0.410
Stigma fear-community	2.77	− 0.1594	− 1.67	0.098
Stigma-avoidant coping	2.37	− 0.0011	− 0.01	0.991
Enacted stigma	7.74	0.0860	0.89	0.374
Internalized stigma	2.76	0.0268	0.28	0.782
Vicarious stigma	2.42	0.0689	0.71	0.477

^*108 observations^

More than half (55.1%) of mother participants had two children. Their children’s ages ranged from 3 to 8 years, but 86% were between ages 6 and 8. Of the total number of children diagnosed with HIV, 55 were male and 54 were female. Their children’s CD4 count (cells/mm^3^) ranged from 258 to 1500 (M = 764, SD = 274.18).

In the unadjusted model, our regression analysis revealed that mother’s stigma-avoidant coping, vicarious stigma, internalized stigma, and enacted stigma were not associated with her child’s CD4 count. Among the stigma fears, the only variable of statistical significance was mother’s community stigma fear. Mother’s community stigma fear was negatively correlated with child CD4 count, where for each one-unit increase in mother’s community stigma fear, her child’s CD4 count decreased by 403 cells (95% CI = − 652, − 155; *t-*value = − 3.22; p = 0.002).

To control for confounding, religion and educational attainment were added to the final model because their p-values (< 0.2) were significant in the univariate analysis. In the adjusted model (Table 3), mother’s community stigma fear remained predictive of child CD4 count. However, we also found an association between maternal religion and child CD4 count. Specifically, mother’s Christian faith was associated with a higher CD4 count in her child.

**Table 3:** Unadjusted and adjusted models (n = 108)

	Base model					Full model				
Variable	*B* Coef	*t-*value	*p*-value	[95% conf. interval]		*B* Coef	*t-*value	*p*-value	[95% conf. interval]	
*Stigma fear*										
Family	49	0.71	0.479	− 88.083	186.511	94	1.29	0.200	− 50.464	238.497
Friends	− 74	− 0.63	0.530	− 306.858	159.043	− 81	− 0.70	0.487	− 311.406	149.384
Healthcare workers	74	0.68	0.501	− 143.487	291.790	48	0.44	0.659	− 168.439	265
Community	− 403	− 3.22	0.002	− 651.752	− 155.159	− 352	− 2.79	0.006	− 602.798	− 102.088
*Stigma-avoidant coping*	54	0.57	0.568	− 131.971	239.312	52	0.55	0.582	− 133.665	236.740
*Enacted stigma*	28	1.38	0.170	− 12.343	68.925	14	0.66	0.511	− 28.554	57.025
*Internalized stigma*	49	0.41	0.686	− 189.077	286.375	40	0.34	0.733	− 194.167	275.093
*Vicarious stigma*	75	1.00	0.320	− 74.060	224.224	80	1.07	0.286	− 68.176	228.358
*Religion*										
Hindu						Ref				
Muslim						118	1.18	0.240	− 80.032	316.160
Christian						125	1.99	0.049	0.487	249.939
*Education*										
None						Ref				
1–9 years						22	0.40	0.692	− 87.897	131.861
≥ 10 years						126	1.45	0.151	− 46.924	298.977

### Association Between Community Stigma Fear and Child CD4 Count

Although data were retrieved for 109 seroconcordant mother–child dyads, our regression analysis (n = 108) reported data for one less dyad due to missing data for mother’s family stigma fear. We examined the association between maternal HIV-related stigma variables and child CD4 as our main objective for this analysis and our adjusted model demonstrated that mother’s community stigma fear was correlated with child CD4 count. Mother’s community stigma fear remained predictive of child CD4 count, demonstrating a negative relationship where for every one-unit increase in mother’s community stigma fear, her child’s CD4 count decreased by 352 cells (95% CI = − 603, − 102; *t*-value = − 2.79; p = 0.006) in the adjusted model.

## Discussion

The literature demonstrating an association between HIV-related stigma and health outcomes has grown significantly [30], and our study further adds to the body of evidence on the association between stigma and health by exploring the correlation between maternal HIV-related stigma and children’s CD4 in rural Indian seroconcordant mother–child dyads, revealing at least one possible relationship. We found a significant negative correlation between community stigma fear and child CD4 count. This finding is consistent with prior studies that show multiple harmful effects of HIV stigma on the health of people living with HIV. Alternatively, our finding is consistent with other literature, suggesting that perhaps having a healthier child shields the mother from fear that she will be rejected by her community. For instance, children living with HIV receive access to health care through parental figures; their diagnostic procedures, medical appointments, and treatment adherence depend on family members’ access to health care services [49]. Our results are in accordance with a previous study [50], that the real or imagined fear may deter mothers from seeking community services and support that would be beneficial to help both mother’s and child’s HIV prognoses. In studies on stigma and care seeking behaviors in Asia [51] and globally [52], stigma has been correlated with barriers to care. We speculate that community stigma fear is correlated with the underutilization of resources and barriers to seeking help, demonstrated by a lower child CD4 count.

In contrast to our finding, Chandy et al. [53] studied a comparable population, WLWH in South India, and found that women who were afraid they would experience community-level stigma were more likely to seek care for their health. However, unlike the Chandy study, we did not look at mothers’ engagement in care, but at their children’s health. It is possible that community-level HIV stigma may have differential impact on seroconcordant mother–child dyads among a broader population of WLWH. For example, fear of blame for the child’s HIV status may increase the harmful effect of community-level HIV stigma on children of women living with HIV. Additional research is needed to elucidate the complex dynamics that affect the relationship between community-level HIV stigma and children’s health.

### Limitations

There were several limitations in our study. The primary limitation is the cross-sectional nature of our study that prevents us from drawing any conclusions regarding causality. Given our study design, it is unknown whether child health influences mother’s stigma fear or mother’s stigma fear influences care seeking and child health. For example, mothers of children who are healthy may be less afraid of community stigma. Longitudinal studies are needed to improve our understanding of whether a causal relationship exists between these variables. Of note, the stigma measures only assessed mother’s fear of how she would be treated if her HIV status was disclosed. The measures did not directly assess mother’s fear of what would happen to her child if her child’s HIV diagnosis was disclosed. It is possible that mother’s stigma fear for herself is different than her fear for her child. Moreover, our study investigated a very specific rural community; therefore, results may not generalize to mothers and children in more urban settings. Lastly, the fact that all children were provided with antiviral therapy as standard practice in India could have affected our correlation estimate. Our results cannot be generalized to families with children who are not on ART as standard practice.

## Conclusions

Our exploratory analysis suggests that mothers who are afraid of stigma from community members are likely to have children with lower CD4 counts. Stronger study designs are needed to improve our understanding of the underlying mechanism for this difference and whether a causal relationship exists between these variables. We emphasize the need for future in-depth qualitative studies and longitudinal quantitative studies that seek to understand the underlying mechanisms whereby stigma impacts clinical outcomes in children. Our findings highlight the need to understand stigma on a more granular level, to better understand the impact of HIV-related stigma on the compounded burden of care in a seroconcordant household where mother and child both live with HIV.

## Data Availability

De-identified data will be shared upon receipt of a completed data request form.

## Code Availability

Study code will be shared upon formal receipt of a completed request form.
